# Case Report: Cryptococcal meningitis in Hodgkin’s Lymphoma patient receiving brentuximab-vedotin therapy

**DOI:** 10.12688/f1000research.24816.2

**Published:** 2020-08-12

**Authors:** Tatiana Cunha Pereira, Rita Rb-Silva, Rita Félix Soares, Nelson Domingues, José Mariz

**Affiliations:** 1 Medical Oncology Department, Instituto Português Oncologia de Coimbra Francisco Gentil E. P. E., Coimbra, Portugal; 2 Onco-Hematology Department, Instituto Português de Oncologia do Porto, Porto, Portugal

**Keywords:** C. neoformans, Brentuximab-vedotin, Hodgkin Lymphoma, Meningitis

## Abstract

*Cryptococcus neoformans* infections occur mostly in immunodeficient individuals, being the most common opportunistic fungal central nervous system (CNS) infection in HIV seropositive patients. Moreover, other conditions affecting host immunity, such as hematologic malignancies, organ transplantation and immunosuppressive drugs are implicated as risk factors.

The authors present a case of a 48-year-old male with Hodgkin Lymphoma for 26 years and submitted to several lines of treatment, diagnosed with cryptococcal meningitis while on therapy with brentuximab. The patient presented with positive cerebral spinal fluid (CSF) cryptococcal antigen plus positive blood cultures. He was put under induction antifungal treatment with liposomal amphotericin B and flucytosine, as well as corticosteroid therapy with dexamethasone with headache improvement and a favorable clinical evolution.

There are no reported cases of cryptococcal meningoencephalitis under CD30-directed monoclonal antibody. Furthermore, this case illustrates the risk of *Cryptococcus neoformans* infection in immunocompromising conditions other than HIV, underlining the need of considering this differential diagnosis when physicians face an opportunistic neuroinfection.

## Learning points

- Cryptococcal meningitis is a common opportunistic central nervous system (CNS) infection among HIV-positive patients. However, it also affects HIV seronegative patients.
- Every immunocompromising condition must be assessed and considered a risk factor for an opportunistic fungal meningoencephalitis. A therapeutic agent affecting host immunity, such as with CD30-directed monoclonal antibody, may predispose to opportunistic infections.
- Cryptococcal meningitis diagnosis may be challenging in cases presenting negative cerebral spinal fluid (CSF) cultures, but cryptococcal polysaccharide antigen titers in CSF correlate with fungal burden.

## Background

Cryptococcus species have a major predilection for the lungs with potential to spread further, mainly through continuity or through hematogenic and lymphoid pathways, with possible penetration through the blood-brain barrier and CNS involvement ^1– 4^.

*Cryptococcus neoformans* infections occur mostly in immunodeficient individuals, being the most common opportunistic CNS infection in HIV-positive patients, counting up to 1 million new infections annually worldwide ^3, 4^. It also occurs in transplant recipients, patients with hematological malignancies, as well as patients receiving immunosuppressive medications ^1, 2, 4^.

This case reports an opportunistic CNS infection in a patient with Hodgkin Lymphoma under brentuximab after multiple lines of treatment for over 20 years, including an allogenic stem cell transplantation. Despite being reported as a common fungal infection in HIV-patients, neuroinfections in patients under CD30-directed monoclonal antibody therapy or other drugs besides immunosuppressants are a rare occurrence.

## Case presentation

A 48-year-old Caucasian male presented at the outpatient clinic in May 2019 with holocranial headache, more intense at occipital level, lasting for 6 days, with increasing intensity over the last couple of hours, associated with photophobia and vomiting.

**Figure 1.**
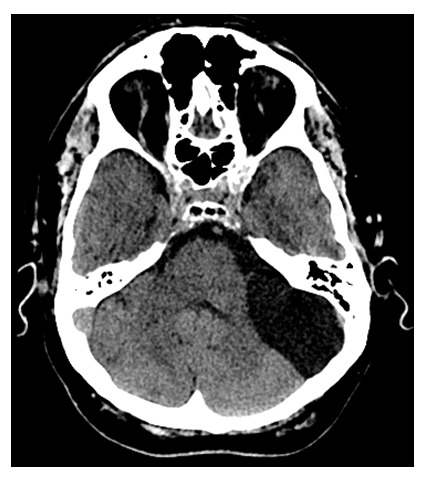
Head computed tomography (CT) scan revealing a large left extra-axial cystic lesion that was being monitored before current symptomatology.

The patient was diagnosed in 1993 with Classic Hodgkin Lymphoma, nodular sclerosis subtype, stage IVB, achieving complete remission after first line chemotherapy. Since then, the patient suffered several relapses and underwent radiotherapy, one autologous bone marrow transplant in 1998, as well as an allogenic stem cell transplant in 2001, followed by several lines of chemotherapy. From October 2018 to this episode, the patient was taking brentuximab due to a hepatic hilar lesion. Sequencial imaging assessments showed a large left infratentorial arachnoid cystic lesion that was being monitored. ( Figure 1).

At first evaluation, the patient was conscious and aware, hemodynamic stable and subfebrile, presenting general tremors and limited cervical mobility.

Blood workup revealed elevated C-reactive protein with 73.2 mg/L (normal range under 5 mg/L), without other abnormalities.

**Figure 2.**
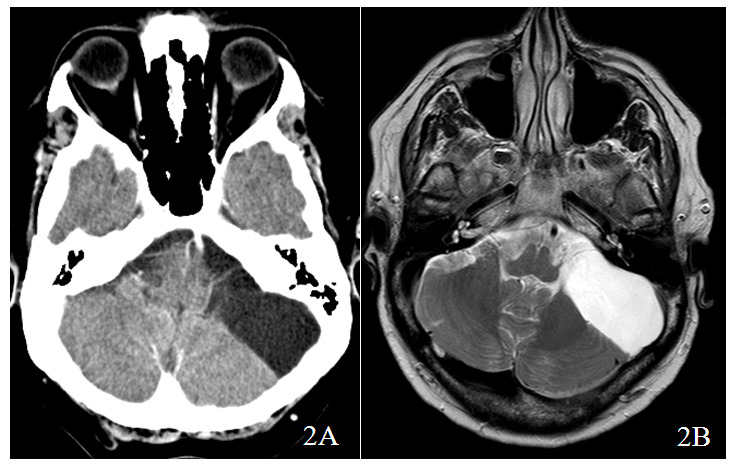
Head computed tomography (CT) scan showed the pre-existing cystic lesion in the left cerebellopontine angle with a slight right brainstem deviation, without associated edema ( **2A**), as confirmed by magnetic resonance imagining (MRI) ( **2B**).

A head computed tomography (CT) scan showed the pre-existing cystic lesion in the left cerebellopontine angle with a slight right brainstem deviation, without associated edema ( Figure 2A), confirmed by magnetic resonance imaging ( Figure 2B). The case was discussed with the Neurosurgery Department and a lumbar puncture was postponed as it was considered a high-risk procedure. The patient started antibiotics with ceftriaxone (2 g q12h) and ampicillin. (2g q4h) At day 4, blood cultures came back positive for *Cryptococcus neoformans* sensitive to Posaconazole, Amphotericin B and Itraconazole, so that patient started Liposomal Amphotericin B (3mg/kg id) and Flucytosine (100 mg/kg per day orally in four divided doses) for 14 days and low dose corticosteroid therapy (4 mg per day). There was a progressive improvement of the symptoms and patient was discharged after 19 days with prescription of Fluconazole (400mg per day).

After one month of treatment, a ventricular puncture was performed and normal pressure cerebrospinal fluid (CSF) revealed glucose consumption and elevated levels of proteins ( Table 1), as well as positivity for cryptococcal polysaccharide capsular antigen. Follow-up lumbar punctures were performed to assess CSF characteristics and cryptococcal antigen assessment. Patient was kept under consolidation therapy with Fluconazole for 10 weeks with a favorable clinical evolution, as well as decreasing levels of protein and nucleated cells count as seen in Table 1. Patient maintains close surveillance under regular appointments at the Onco-Haematology Clinic. However, headache complaints increased in intensity shortly after dexamethasone discontinuation with an intermittent pattern. Patient died in another hospital about 8 months after the meningitis diagnosis due to a cardiovascular event.

**Table 1.** Cerebrospinal fluid profile evolution throughout treatment. CSF – Cerebrospinal fluid. LP – Lumbar puncture. NV – Normal value.

LP date	27-06-2019	17-07-2019	31-07-2019	16-09-2019
Characteristic				
**Appearance**	Clear	Clear	Clear	Clear
**Nucleated cells count**	104/μL	43/μL	35/μL	5/μL
**Glucose** **(NV: 2.8 – 4.4 mmol/L)**	2,3 mmol/L	3.1 mmol/L	3,4 mmol/L	3,3 mmol/L
**Protein level** **(NV: 150 – 450 mg/L)**	838 mg/L	583 mg/L	529 mg/L	544 mg/L
**CSF culture**	Negative	Negative	Negative	Negative
**Cryptococcus neoformans** **antigen**	Positive	Positive	Positive	Positive

## Discussion

Cryptococcal meningitis accounts for up to 1 million new infections annually, mainly affecting HIV-positive patients. Other immunocompromising conditions such as organ transplantation, hematologic malignancies and immunosuppressive drugs constitutes other relevant risk factors to these opportunistic fungi CNS infections ^1– 4^.

In a recent review of *Cryptococcus neoformans* infections in patients with cancer, 82% corresponded to patients with haematological malignancies and from these patients, approximately 54% had lymphoma ^5^.

The patient presented several conditions affecting host immunity due to several previous lines of treatment for over 25 years. However, Cryptococcus species were not considered the etiological agent for a possible opportunistic neuroinfection, emphasizing the need for an initial lumbar puncture to exclude fungal agents. This procedure was not possible at first evaluation and it delayed the start of antifungal therapy.

Although there are many published case reports of Cryptococcosis in patients with lymphoma, this is the first reported case of Cryptococcal neuroinfection in a patient with Hodgkin’s Lymphoma treated with CD-30-directed monoclonal antibody.

## Consent

Written informed consent for publication of their clinical details and clinical images was obtained from the patient prior to their death.

## Data availability

### Underlying data

All data underlying the results are available as part of the article and no additional source data are required.
