# Supplementary material for: Case Report: Cryptococcal meningitis in Hodgkin’s Lymphoma patient receiving brentuximab-vedotin therapy
Source: F1000Res. 2020 Aug 12;9:687. Originally published 2020 Jul 8. [Version 2] doi: 10.12688/f1000research.24816.2 (PMC7512028; doi:10.12688/f1000research.24816.2)
Supplement: Peer review history

# Peer review: Reviewer response for version 2

**DOI:** 10.5256/f1000research.28541.r69298

**Published:** 2020-09-23

**Authors:** Sarah A. Schmalzle

**Affiliations:** 1 Institute of Human Virology, University of Maryland School of Medicine, Baltimore, MD, USA

No new comments.

Are enough details provided of any physical examination and diagnostic tests, treatment given and outcomes?

Yes

Is the case presented with sufficient detail to be useful for other practitioners?

Yes

Is sufficient discussion included of the importance of the findings and their relevance to future understanding of disease processes, diagnosis or treatment?

Yes

Is the background of the case’s history and progression described in sufficient detail?

Yes

Reviewer Expertise:

Infectious disease, HIV, cryptococcosis, Group A Strep

I confirm that I have read this submission and believe that I have an appropriate level of expertise to confirm that it is of an acceptable scientific standard.

# Peer review: Reviewer response for version 1

**DOI:** 10.5256/f1000research.27380.r68025

**Published:** 2020-07-30

**Authors:** Sarah A. Schmalzle

**Affiliations:** 1 Institute of Human Virology, University of Maryland School of Medicine, Baltimore, MD, USA

This is a clinically relevant case report as it is purportedly the first report in a patient being treated with a particular immunosuppressive monoclonal Ab. 

 There are several minor language and grammatical improvements to be made that will strengthen and clarify the report. It should have an additional round of editing by a native English speaker. Examples: 

1. HIV-positive should be 'people living with HIV'.
2. Abstract: 'fungi central nervous system..' should be fungal.
3. Brentuximab should be in lower case throughout. It is not explained with it is 'brentuximab-vedotin' in keywords but 'brentuximab' elsewhere.
4. End of abstract - opportunist should be opportunistic. 
5. This sentence needs an English language revision: "However, these fungi neuroinfection affects HIV seronegative patients." 
6. Background - it is not accurate to say crypto has a predilection for the lungs. The lungs are the route of entry, but this is a neurotropic pathogen. 
7. 'penetration into' should be 'penetration through'.
8. Case presentation: "over the last couple hours" should be "over the last couple of hours" or "over the last several hours".
9. The use of 'under' to describe being prescribed a treatment is not commonly how this is stated in the US. Try "treated with" or "on treatment for ___ with ___".
10. Also not common to say patients are admitted at the outpatient clinic. Patients are seen or evaluated at a clinic, but admitted to a hospital. 
11. This sentence also needs to be revised for proper grammar/syntax. "Throughout image assessments, a large left infratentorial arachnoid cystic lesion was being monitored.".
12. Susceptibilities are not commonly reported in Crypto case reports. There is not known resistance and generally all therapy is empiric.
13. Corticotherapy should be corticosteroid therapy.
14. This needs to be reworded for clarity: "However, Cryptococcus species were not considered the etiological agent for a CNS infection, emphasizing the need for an initial lumbar puncture to exclude fungal agents when approaching opportunist neuroinfection. This was not possible at first evaluation in this case which delayed antifungal therapy."

Are enough details provided of any physical examination and diagnostic tests, treatment given and outcomes?

Yes

Is the case presented with sufficient detail to be useful for other practitioners?

Yes

Is sufficient discussion included of the importance of the findings and their relevance to future understanding of disease processes, diagnosis or treatment?

Yes

Is the background of the case’s history and progression described in sufficient detail?

Yes

Reviewer Expertise:

Infectious disease, HIV, cryptococcosis, Group A Strep

I confirm that I have read this submission and believe that I have an appropriate level of expertise to confirm that it is of an acceptable scientific standard, however I have significant reservations, as outlined above.

# Peer review: Reviewer response for version 1

**DOI:** 10.5256/f1000research.27380.r66704

**Published:** 2020-07-20

**Authors:** Adriana Roque

**Affiliations:** 1 Clinical Hematology Department, Centro Hospitalar e Universitário de Coimbra, Coimbra, Portugal; 2 Faculty of Medicine, Center for Innovative Biomedicine and Biotechnology (CIBB), Coimbra Institute for Clinical and Biomedical Research (iCBR), Coimbra, Portugal

I think that this is an interesting report that claims for attention to rare infections in patients under immunotherapy and other novel therapies, especially when they are more difficult to diagnose (due to the location and the infectious agent).

 This report provides a concise and informative history of the case, mainly focus on the difficulties of the process.

 I consider that it would be important to provide information about the previous therapeutics that the patient had received, including the transplantation conditioning regimen, as well as the development of graft versus host disease (GvHD) and GvHD therapeutics, because it can help to explain the subjacent immunosuppression state. Another helpful information is to understand if Hodgkin lymphoma was under control at the time of CNS infection presentation.

Are enough details provided of any physical examination and diagnostic tests, treatment given and outcomes?

Yes

Is the case presented with sufficient detail to be useful for other practitioners?

Yes

Is sufficient discussion included of the importance of the findings and their relevance to future understanding of disease processes, diagnosis or treatment?

Yes

Is the background of the case’s history and progression described in sufficient detail?

Partly

Reviewer Expertise:

Hematology, Hematology-oncology

I confirm that I have read this submission and believe that I have an appropriate level of expertise to confirm that it is of an acceptable scientific standard.
